# Fine Particulate Matter Pollution and Risk of Community-Acquired Sepsis

**DOI:** 10.3390/ijerph15040818

**Published:** 2018-04-21

**Authors:** Elisa J. Sarmiento, Justin Xavier Moore, Leslie A. McClure, Russell Griffin, Mohammad Z. Al-Hamdan, Henry E. Wang

**Affiliations:** 1 Department of Emergency Medicine, University of Alabama at Birmingham, Birmingham, AL 35233, USA; esarmiento@uabmc.edu (E.J.S.); jxmoore@wustl.edu (J.X.M.); 2 Department of Epidemiology, University of Alabama at Birmingham, 1665 University Boulevard, RPHB, Birmingham, AL 35233, USA; rlgriffin@uabmc.edu; 3 Division of Public Health Sciences, Department of Surgery, Washington University in Saint Louis School of Medicine, St. Louis, MO 63110, USA; 4 Department of Epidemiology and Biostatistics, Drexel University, Philadelphia, PA 19104, USA; lam439@drexel.edu; 5 Universities Space Research Association, NASA Marshall Space Flight Center, Huntsville, AL 35805, USA; mohammad.alhamdan@nasa.gov; 6 Department of Emergency Medicine, University of Texas Health Science Center at Houston, 6431 Fannin St., JJL 434, Houston, TX 77030, USA

**Keywords:** sepsis, air pollution, particulate matter

## Abstract

While air pollution has been associated with health complications, its effect on sepsis risk is unknown. We examined the association between fine particulate matter (PM_2.5_) air pollution and risk of sepsis hospitalization. We analyzed data from the 30,239 community-dwelling adults in the Reasons for Geographic and Racial Differences in Stroke (REGARDS) cohort linked with satellite-derived measures of PM_2.5_ data. We defined sepsis as a hospital admission for a serious infection with ≥2 systemic inflammatory response (SIRS) criteria. We performed incidence density sampling to match sepsis cases with 4 controls by age (±5 years), sex, and race. For each matched group we calculated mean daily PM_2.5_ exposures for short-term (30-day) and long-term (one-year) periods preceding the sepsis event. We used conditional logistic regression to evaluate the association between PM_2.5_ exposure and sepsis, adjusting for education, income, region, temperature, urbanicity, tobacco and alcohol use, and medical conditions. We matched 1386 sepsis cases with 5544 non-sepsis controls. Mean 30-day PM_2.5_ exposure levels (Cases 12.44 vs. Controls 12.34 µg/m^3^; *p* = 0.28) and mean one-year PM_2.5_ exposure levels (Cases 12.53 vs. Controls 12.50 µg/m^3^; *p* = 0.66) were similar between cases and controls. In adjusted models, there were no associations between 30-day PM_2.5_ exposure levels and sepsis (4th vs. 1st quartiles OR: 1.06, 95% CI: 0.85–1.32). Similarly, there were no associations between one-year PM_2.5_ exposure levels and sepsis risk (4th vs. 1st quartiles OR: 0.96, 95% CI: 0.78–1.18). In the REGARDS cohort, PM_2.5_ air pollution exposure was not associated with risk of sepsis.

## 1. Background

Sepsis is a dangerous syndrome of systemic inflammation triggered by microbial infection such as pneumonia, kidney infection, cellulitis, and meningitis [1]. Sepsis may rapidly lead to organ injury, shock, and death. Sepsis is a major threat to U.S. community health, resulting in over 750,000 hospitalizations and 215,000 deaths in the USA annually—more than from acute myocardial infarction, lung cancer, or breast cancer [2]. 

Environmental exposures such as PM_2.5_ (fine particulate matter with diameter equal to or smaller than 2.5 µm) have been associated with a host of health risks including increased rates of cardiovascular disease, pneumonia, asthma, stroke, and all-cause mortality [3,4,5,6,7,8,9]. There are plausible connections between environmental exposures and sepsis risk. Inflammation and vascular endothelial dysfunction are prominent in sepsis [10]. PM_2.5_ directly enters the bloodstream, causing similar systemic inflammation and vascular damage [4]. Prior research links long-term PM_2.5_ exposure with a 2.3-fold increased pneumonia hospitalization risk [7]. Of note, using U.S. vital statistics, we observed there to be a belt of states located in the Southeastern USA “belt” with a more than two-fold increased risk of sepsis mortality [11].

To date, no studies have examined whether long-term exposure to PM_2.5_ is associated with increased risk of sepsis. The objective of this study was to determine whether short- or long-term exposure to PM_2.5_ is associated with the incidence of sepsis in a large national community-dwelling cohort.

## 2. Methods

### 2.1. Study Design 

We performed a nested case-control study within the Reasons for Geographic and Racial Differences in Stroke (REGARDS) cohort, an ongoing national longitudinal cohort. This study was approved by the University of Alabama at Birmingham Institutional Review Board (UAB IRB protocol X090531004).

### 2.2. Data Source

Designed to evaluate factors associated with racial and geographic differences in stroke mortality, the REGARDS cohort consists of 30,239 community-dwelling adults aged ≥45 years at baseline [12]. Among REGARDS participants, approximately 45% are male, 41% are black, and 69% are aged >60 years. REGARDS recruited participants between January 2003 and October 2007. At six-month intervals, REGARDS contacts participants by telephone to identify any hospitalizations. Further details for the REGARDS study methods are described elsewhere [13]. 

### 2.3. Selection of Sepsis Cases

The primary exposure in this study was defined as first hospitalization for sepsis between 1 January 2003 and 31 December 2011. Using the taxonomy of Angus et al., we identified all hospitalizations (Emergency Department visits and/or hospital admission) attributed by participants to a serious infection [2,14]. We defined sepsis cases as hospital admission for a serious infection with the presence of at least two Systemic Inflammatory Response Syndrome (SIRS) criteria. The SIRS criteria included: (1) heart rate >90 beats/min; (2) fever (temperature >38.3 °C or <36.0 °C); (3) tachypnea (>20 breaths/min) or PCO2 <32 mmHg; and (4) leukocytosis or leukopenia (white blood cells >12,000 or <4000 cells/mm^3^ or >10% band forms) [13,15]. Since our study focused on community-acquired (vs. hospital-acquired) sepsis, we used vital signs and laboratory test results for the initial 28 h of hospitalization. Two trained reviewers evaluated information from the corresponding medical record, confirming the presence of a serious infection based upon diagnoses documented in the Emergency Department or admission physician record. Inter-rater agreement for infection (*k* = 0.92) and presence of sepsis (*k* = 0.90) upon hospital presentation was excellent. Discordances were adjudicated among abstractors, with additional physician review as needed. 

### 2.4. Selection of Matched Controls

**Figure 1:**
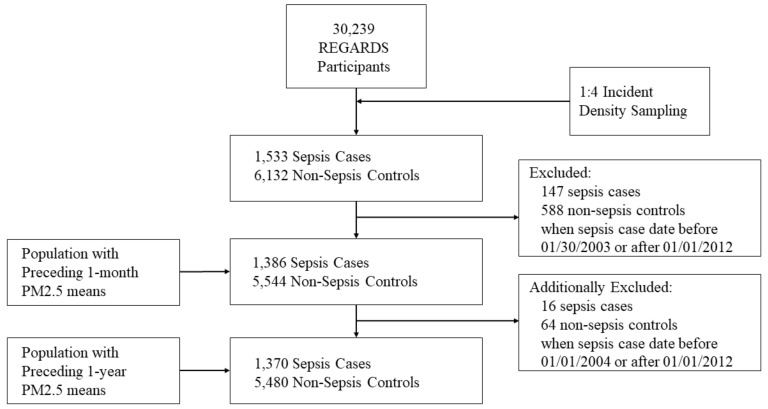
Breakdown of REGARDS participants used in nested case-control study for the association between short-term PM_2.5_ exposure and sepsis.

We performed incidence density sampling matching of 1533 sepsis cases with (6132 non-sepsis controls by age (±5 years), sex, and race (Figure 1). The incidence density sampling method matched each sepsis case with four controls based upon the sepsis case event date. Controls were participants that were alive for at least the same time preceding the sepsis case event but without a sepsis event or death. For example, if a sepsis case event were on 31 January 2008 this sepsis case was matched with four controls (of similar age (±5 years), sex, and race) from the entire study cohort that had not died, dropped out, or had a sepsis event up to that date. This matching prevents large imbalance of cases versus controls and optimizes the statistical efficiency by controlling for possible confounding (i.e., age, sex, race) [16]. Cases and controls were selected with replacement; sepsis cases could also be selected as a matched control for another sepsis cases during their time preceding their sepsis event. In addition, non-sepsis controls could serve as a matched control for more than one sepsis case. 

### 2.5. Measurement of Exposure (PM_2.5_)

The primary exposures of interest were average prior 30-day and one-year daily PM_2.5_ for the residence of each REGARDS participant. We followed the method of Al-Hamdan et al., generating continuous spatial surfaces of daily PM_2.5_ on a 10-km grid for the entire contiguous US for years 2003 through 2011 [17]. These data were derived from the U.S. Environmental Protection Agency Air Quality System (AQS) and the NASA Moderate Resolution Imaging Spectrometer (MODIS) instrument onboard the Aqua Earth-orbiting satellite. Leveraging NASA MODIS-derived data to complement U.S. EPA ground observation data, Al-Hamdan et al. estimated daily PM_2.5_ concentrations using regional spatial surfacing algorithm, which included regression models, a B-spline smoothing model, a quality control procedure for the EPA AQS data and a bias adjustment procedure for MODIS/Aerosol Optical Depth-derived PM_2.5_ data [17,18]. The surfacing algorithm resulted in continuous spatial surfaces of daily PM_2.5_ on a 10-km grid for the contiguous USA from the combination of AQS PM_2.5_ measurements and the MODIS-estimated PM_2.5_. Merging MODIS remote sensing data with surface observations of PM_2.5_ not only provided a more complete daily representation of PM_2.5_ than either dataset alone would allow, but it also reduced the errors in the PM_2.5_-estimated surfaces [17,18].

We estimated daily PM_2.5_ concentrations for the geocoded location of REGARDS participants from January 2003 through December 2011. We then estimated the 30-day and one-year daily mean PM_2.5_ concentrations preceding the matched sepsis event date (days leading up to the matched sepsis event date). We excluded subjects where exposure data were not available prior to REGARDS enrollment. For statistical analyses, we categorized both 30-day and one-year PM_2.5_ as a categorical variable by quartiles and we standardized using the standard deviation of the study population. 

### 2.6. Participant Characteristics

Participant characteristics used in the analysis included self-reported age, race, sex, income, education, and geographic location. Health behaviors included tobacco (defined as current, past and never) and alcohol use. We defined alcohol use as moderate (one drink per day for women or two drinks per day for men) and heavy alcohol use (>1 drink per day for women and >2 drinks per day for men), per the National Institute on Alcohol Abuse and Alcoholism classification [15]. 

We included atrial fibrillation, chronic lung disease, coronary artery disease, deep vein thrombosis, diabetes, dyslipidemia, hypertension, myocardial infarction, obesity, peripheral artery disease, and stroke as baseline medical conditions. We defined atrial fibrillation by participant self-report or baseline electrocardiogram (ECG) evidence of an atrial fibrillation event. We defined chronic lung disease as participants with a history of prescribed pulmonary medication. We classified coronary artery disease in participants with a history of heart disease (self-reported myocardial infarction, coronary artery bypass graft, bypass, angioplasty, or stent) or ECG evidence. We defined diabetes as a fasting glucose ≥126 mg/L (or a glucose ≥200 mg/L for those not fasting) or the use of insulin or oral hypoglycemic agents. We categorized dyslipidemia in participants with low-density lipoprotein cholesterol >130 mg/dL, or use of lipid lowering medications. We defined hypertension as a systolic blood pressure ≥140 mm Hg, diastolic blood pressure ≥90 mm Hg, or the reported use of antihypertensive agents. We defined myocardial infarction (MI) in participants by ECG evidence or self-reports of MI in participants’ history. Obesity was defined as a body mass index (BMI) of greater than or equal to 30 kg/m^2^ in addition to a gender specific waist circumference [17]. 

REGARDS did not collect information on pulmonary conditions such as asthma and chronic obstructive pulmonary disease. Therefore, we defined participant use of pulmonary medications as a proxy for chronic lung disease. Obtained from each participant’s medication inventory, pulmonary medications included beta-2 adrenergic agonists, leukotriene inhibitors, inhaled corticosteroids, combination inhalers, and other pulmonary medications such as ipratropium, cromolyn, aminophylline, and theophylline. Other medical conditions such as; deep vein thrombosis, peripheral artery disease, and stroke were based upon self-reports. Detailed definitions of participant characteristics are described in Appendix Table A1.

### 2.7. Community Characteristics

Community characteristics analyzed in this study included season and urbanicity. We defined season based on the REGARDS participants’ baseline interview date. We categorized participants’ baseline season using meteorological definitions for spring (1 March–31 May), summer (1 June–31 August), fall (1 September–30 November), and winter (1 December–28 or 29 February) [19]. This method also allows for an even distribution of calendar days per season [19]. We identified the urbanicity of each participant’s baseline residence using the county-level proportion of population that lived in an urban area using data from the 2010 American Community Survey (ACS). The 2010 ACS is five-year aggregated (2006–2010) data based on a representative sample of non-institutionalized US residents [20]. The 2010 ACS defined urbanicity based on the primary Rural-Urban Commuting Area (RUCA) codes (10-tier classification system). The 10 RUCA code classification system was dichotomized into (1) urban (i.e., population centers with 50,000 or more residents) and (2) non-urban (i.e., towns or small cities with population centers with less than 50,000 residents [21,22]. Based on the proportion of residents living in urban counties we then categorized REGARDS participants as living in either a rural (≤25% urban population), mixed (25–75% urban population), or urban county (≥75% urban population).

### 2.8. Statistical Analysis

We compared baseline sociodemographic, community characteristics, health behaviors, and chronic medical conditions between sepsis cases and non-sepsis controls using conditional logistic regression for categorical variables and paired *t*-test for continuous variables. We fit two different models to examine association between preceding 30-day mean and one-year mean PM_2.5_ and the odds of sepsis, separately. First, we evaluated the associations between preceding 30-day mean PM_2.5_ and development of sepsis using conditional logistic regression, categorizing the preceding 30-day mean PM_2.5_ per standard deviation. Secondly, we evaluated the associations between preceding 30-day mean PM_2.5_ and development of sepsis using conditional logistic regression, categorizing the preceding 30-day mean PM_2.5_ as quartiles with the first quartile as the referent group. We fitted similar models to examine the associations between one-year mean PM_2.5_ and the future development of sepsis. We sequentially adjusted the models for variables observed to be statistically different on bivariate analysis, including: (1) sociodemographic and environmental variables (i.e., education, income, region, temperature, and urbanicity); (2) health behaviors (tobacco and alcohol use); and (3) baseline medical conditions. We performed statistical analysis using SAS version 9.4 and STATA version 13. 

## 3. Results

Among the 30,239 REGARDS participants, we created a 4:1 matched cohort consisting of 1533 sepsis cases and 6132 non-sepsis controls (Figure 1).

We excluded observations with sepsis case matched dates before 30 January 2003 and after 31 December 2011 due to unavailable PM_2.5_ daily estimates. For the 30-day exposure analysis, we included 1386 cases and 5544 non-sepsis controls. For the one-year exposure analysis, we included 1370 cases and 5480 non-sepsis controls. 

Compared with the 5544 non-sepsis controls, sepsis cases had lower education and income, were more likely to be smokers, and were more likely to have a number of comorbidities at baseline (Table 1).

**Figure 2:**
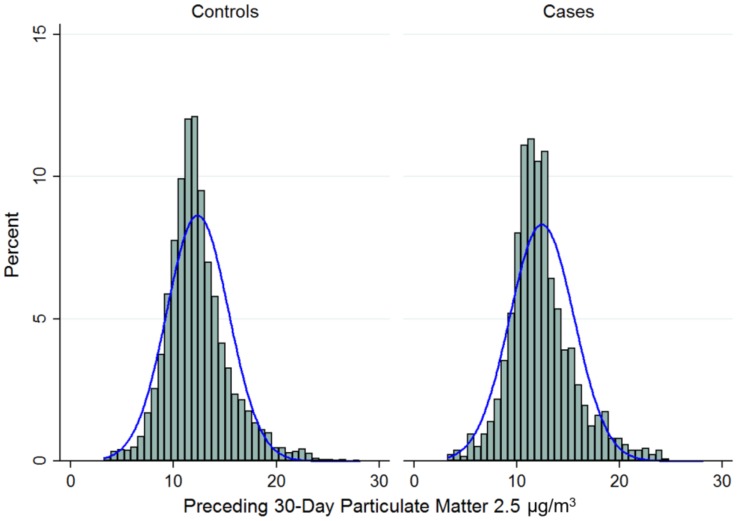
Distribution of average daily mean particulate values for 30-day preceding sepsis case event among 6906 cases and controls.

Mean PM_2.5_ exposure levels were similar between cases and controls at 30 days (Cases: 12.44 (SD 3.13) vs. Controls: 12.34 (3.02) µg/m^3^; *p* = 0.28) and one-year (Cases: 12.53 (1.73) vs. Controls: 12.50 (1.73) µg/m^3^; *p* = 0.66) (Figure 2).

When examining the associations between preceding 30-day PM_2.5_ exposure and sepsis, higher PM_2.5_ levels were not associated with adjusted odds of sepsis (2nd vs. 1st quartile OR 0.93 [95% CI: 0.76–1.14]; 3rd vs. 1st quartile OR 0.86 [95% CI: 0.70–1.05]; 4th vs. 1st quartile OR 1.06 [95% CI: 0.85–1.32]) (Table 2). 

When examining the associations between preceding one-year mean PM_2.5_ and sepsis, higher levels of PM_2.5_ were not associated with odds of sepsis, even after adjustment for potential confounders (2nd vs. 1st quartile OR 0.99 [95% CI: 0.82–1.21]; 3rd vs. 1st quartile OR 1.04 [95% CI: 0.85–1.27]; 4th vs. 1st quartile OR 0.96 [95% CI: 0.78–1.18]).

We observed similar results when limiting the analysis to respiratory-infection-related sepsis events only (Appendix Table A2).

## 4. Discussion

In this study, we observed no associations between 30-day and one-year PM_2.5_ exposure and future risk of odds of community-acquired sepsis. Prior studies have linked air pollution with the risk of infections and other medical conditions. For example, in a 2009 case-control study Neupane et al. reported that among individuals aged 65 years or older, there was a 2-fold increased risk of hospitalization for community-acquired pneumonia in those exposed to elevated levels of ambient nitrogen and PM_2.5_ [7]. Further, a recent meta-analysis by Brook et al. concluded that PM_2.5_ exposure can prompt cardiovascular disease and that long-term exposure may increase the risk for cardiovascular mortality. Brook et al. also concluded that decreased life expectancy could be attributed to PM_2.5_ in highly exposed populations [3]. Lastly, in a 2004 cohort study, Pope et al. examined PM_2.5_ exposure and the risk of mortality related to cardiovascular conditions, including ischemic heart disease, dysrhythmias, hypertension, atherosclerosis, and diabetes. Pope et al. reported that individuals with increased PM_2.5_ exposure had an elevated risk of mortality from combined cardiovascular causes plus diabetes (OR 1.12 [95% CI: 1.08–1.15) [9].

We originally hypothesized that PM_2.5_ exposure would be linked with sepsis risk due to the known associations of particulate matter exposure with infection risk and induction of cardiopulmonary toxicity [23]. It is plausible that other environmental factors were not accounted for in our analysis. For example, other environmental exposures such as solar insulation or geographical altitude could have contributed to the observed effects of particulate matter on sepsis risk. Individuals living at higher attitudes may have had lower PM_2.5_ tolerance due to lower atmospheric oxygenation. Most importantly, our study used U.S. data. Exposure patterns and associations may differ in other countries with varying populations, pollution sources and climate. Future studies may consider prospective pollution measurements obtained from on-person or in-home air quality monitors.

After controlling for potential risk factors for sepsis such as multiple baseline medical conditions, alcohol use, and tobacco use, there were no significant differences. However, our prior studies have identified a number of individual level risk factors strongly associated with sepsis among community dwelling adults [10,24,25]. The overarching aim of these studies is to identify opportunity to prevent or reduce long-term sepsis risk. We previously identified regional variations in sepsis mortality. Regional variations may be due to a range of factors including geographic similarities in comorbid burden, healthcare quality and environmental exposures. Given the geographic similarities between U.S. sepsis mortality and PM_2.5_ patterns, we expected that PM_2.5_ environment exposure might alter sepsis risk. However, the data from this research does not support this supposition. Efforts to reduce community sepsis risk should focus on other factors.

### Limitations

The REGARDS-sepsis cohort provides the unique opportunity to examine the risk of sepsis after exposure to community-level fine particulate matter exposure among a cohort of community-dwelling adults; however, the results of this analysis should be interpreted with a few limitations in mind. First, the original REGARDS cohort relied upon participant self-report of hospital admission within the past six months. Therefore, it is plausible that due to non-differential misclassification we observed an underestimate of the true number of sepsis cases. A future study should aim to prospectively examine sepsis cases among a large nationally representative sample of community-dwelling adults. The REGARDS cohort included participants 45 and older at baseline and therefore the results of this study are not generalizable to the younger U.S. adult population. In addition, the PM_2.5_ data in this analysis were based upon a combination of satellite and ground-level data; different observations may have resulted from direct PM_2.5_ sampling. Measured particulate matter exposure may not represent the participants’ physiological exposure. REGARDS is a national cohort but not nationally representative. The participants’ baseline geocoded residences may not reflect an accurate estimate of individual particulate matter exposure due to the possibility of the participants’ non-residential environments. Participant migration may have altered the assumed PM_2.5_ exposures. Nevertheless, to our knowledge no prior study has evaluated the associations between sepsis and air pollution. In addition, our study provides great generalizability and addresses a gap within the research literature because we utilized one of the largest United States population-based cohorts complemented with NASA national particulate matter data. 

## 5. Conclusions

In the REGARDS cohort, PM_2.5_ air pollution exposure was not associated with odds of sepsis. While we identified no association between air pollution and sepsis, future studies should aim to assess individual-level air pollution exposure with time-varying measurements.

## Figures and Tables

**Table 1:** Comparison of demographic, substance use, and comorbidity characteristics between sepsis cases and controls. Among 1386 sepsis cases and 5544 controls matched on age (±5 years), race, and sex.

	Sepsis Cases (*n* =1386)	Non-Sepsis Controls * (*n* = 5544)	*p* value **
**Age ^†^**	68.2 (9.3)	67.9 (9.1)	-
**Sex (%)**			
Male	714 (51.5)	2856 (51.5)	-
Female	672 (48.5)	2688 (48.5)	
**Race (%)**			
Black	463 (33.4)	1852 (33.4)	-
White	923 (66.6)	3692 (66.6)	
**< High School Education (%)**	231 (16.7)	580 (10.5)	<0.01
**Income ≤ $20 000 (%)**	332 (24.0)	872 (15.7)	<0.01
**Geographic Region (%)**			
Stroke Belt ^a^	527 (38.0)	1833 (33.1)	<0.01
Buckle ^b^	294 (21.2)	136 (20.5)	
Non-Stroke Belt ^c^	565 (40.8)	2575 (46.5)	
**Temperature 30-Day Mean (°F) ^€^**	59.5 (48.6–65.5)	59.1 (47.4–72.8)	<0.01
**Temperature 365-Day Mean (°F) ^€^**	62.4 (56.7–65.7)	61.9 (54.9–65.6)	0.03
**Season**			1.0
Fall	301 (21.7)	1204 (21.7)	
Spring	371 (26.8)	1484 (26.8)	
Summer	309 (22.3)	1236 (22.3)	
Winter	405 (29.2)	1620 (29.2)	
**Urbanicity (%)**			
Mixed	455 (32.9)	1582 (28.6)	<0.01
Rural	84 (6.1)	278 (5.0)	
Urban	844 (61.0)	3670 (61.0)	
**Tobacco Use (%)**			
Never	485 (35.2)	2539 (46.0)	<0.01
Past	648 (47.0)	2364 (42.8)	
Current	245 (17.8)	619 (11.2)	
**Alcohol Use (%)**			
Never	916 (67.5)	3341(61.4))	<0.01
Past	390 (28.7)	1846 (34.0)	
Current	52 (3.8)	251 (4.6)	
**Baseline Medical Condition (%)**			
Atrial fibrillation	185 (13.6)	499 (9.2)	<0.01
Chronic lung disease	263 (19.0)	453 (8.2)	<0.01
Coronary artery disease	390 (28.9)	1089 (20.0)	<0.01
Deep vein thrombosis	114 (8.3)	295 (5.3)	<0.01
Diabetes	447 (32.3)	1163 (21.0)	<0.01
Dyslipidemia	865 (65.1)	3242 (60.4))	<0.01
Hypertension	945 (68.5)	3261 (59.0)	<0.01
Myocardial infarction	279 (20.6)	760 (14.0)	<0.01
Obesity	846 (61.2)	2718 (49.1)	<0.01
Peripheral artery disease	64 (4.6)	94 (1.7)	<0.01
Stroke	150 (10.9)	347 (6.3)	<0.01

* Matched for age, sex, and race, **^†^** Mean (Standard deviation), **^€^** Median (IQR); ** Estimated using conditional logistic regression (Wald test) or paired *t*-test. ^a^ Stroke Buckle (coastal plains of North Carolina, South Carolina, and Georgia). ^b^ Stroke Belt (remainder of North Carolina, South Carolina and Georgia, plus Tennessee, Mississippi, Alabama, Louisiana, and Arkansas). ^c^ Non-Belt/Buckle (other states).

**Table 2:** Odds ratios * (ORs) and associated 95% confidence intervals (CIs) for the association between the preceding 30-day and one-year mean particulate matter 2.5 and incident sepsis. Among 1386 sepsis cases and 5544 controls matched on age (±5 years), race, and sex.

	Unadjusted	Model 1 ^a^	Model 2 ^b^	Model 3 ^c^
	Preceding 30-Day Mean OR (95% CI)			
**Particulate Matter 2.5**				
Per SD µg/m^3^ increase (SD** = 3.04)	1.06 (0.98, 1.13)	1.04 (0.96, 1.12)	1.03 (0.96, 1.11)	1.04 (0.96, 1.13)
*p*-Value	0.13	0.32	0.40	0.36
**Particulate Matter 2.5**				
1st Quartile (3.27–10.53 µg/m^3^)	Referent	Referent	Referent	Referent
2nd Quartile (10.53–11.97 µg/m^3^)	1.05 (0.88, 1.25)	0.98 (0.82, 1.17)	0.98 (0.82, 1.18)	0.93 (0.76, 1.14)
3rd Quartile (11.97–13.78 µg/m^3^)	0.97 (0.82, 1.16)	0.91 (0.76, 1.09)	0.90 (0.75, 1.09)	0.86 (0.70, 1.05)
4th Quartile (13.78–28.11 µg/m^3^)	1.14 (0.94, 1.38)	1.09 (0.89, 1.32)	1.08 (0.88, 1.32)	1.06 (0.85, 1.32)
*p*-value_trend_	0.36	0.31	0.31	0.22
	**Preceding One-Year Mean OR (95% CI)**			
**Particulate Matter 2.5**				
Per SD µg/m^3^ increase (SD** = 1.73)	1.03 (0.96, 1.09)	0.99 (0.92, 1.06)	0.98 (0.92, 1.05)	0.98 (0.91, 1.05)
*p*-value	0.45	0.71	0.59	0.56
**Particulate Matter 2.5**				
1st Quartile (5.88–11.48 µg/m^3^)	Referent	Referent	Referent	Referent
2nd Quartile (11.48–12.58 µg/m^3^)	1.10 (0.93, 1.30)	1.00 (0.84, 1.19)	0.99 (0.82, 1.18)	0.99 (0.82, 1.21)
3rd Quartile (12.58–13.53 µg/m^3^)	1.15 (0.97, 1.36)	1.05 (0.88, 1.26)	1.05 (0.87, 1.26)	1.04 (0.85, 1.27)
4th Quartile (13.53–20.18 µg/m^3^)	1.10 (0.92, 1.32)	1.00 (0.83, 1.21)	0.98 (0.81, 1.26)	0.96 (0.78, 1.18)
*p*-value_trend_	0.44	0.92	0.88	0.87

* Estimated using Conditional Logistic Regression. ** Standard Deviation (SD). ^a^ Additionally adjusted education, income, region, temperature (30-day or 365 depending on pollution exposure), and urbanicity. ^b^ Additionally adjusted for tobacco and alcohol use. ^c^ Additionally adjusted for baseline health conditions.

## Appendix A.

**Table A1:** Detailed definitions and technical information for sociodemographics, health behaviors, and chronic medical conditions.

Characteristics	Definition and/or Technical Information
**Sociodemographics**	
Age	Age in years
Gender	Male; female
Race	African-American; white
Education	Participant reported: - Less than high school - High school graduate - Some college - College or higher - Missing
Income	Participant reported: - <$20 k - $20–$34K - $35–$74K - ≥$75K - Missing (not reported)
Geographic Region	Participant residence: - Stroke Buckle (coastal plains of North Carolina, South Carolina, and Georgia) - Stroke Belt (remainder of North Carolina, South Carolina, and Georgia, plus Tennessee, Mississippi, Alabama, Louisiana, and Arkansas) - Non-Belt/Buckle (other states)
**Health Behaviors**	
Smoking Status	Participant reported: - Current - Past - Never
Alcohol use	Participant reported: - None - Moderate (up to one drink per day for women or two drinks per day for men) - Heavy (>1 drink per day for women and >2 drinks per day for men) [26].
**Chronic Medical Conditions**	
Atrial Fibrillation	Participant reported history of atrial fibrillation.
Chronic Lung Disease	Participant use of pulmonary medications (beta agonists, leukotriene inhibitors, inhaled corticosteroids, combination inhalers, ipratropium, cromolyn, aminophylline and theophylline) as a surrogate for chronic lung disease.
Coronary Artery Disease	Participant reported history of myocardial infarction, coronary artery bypass grafting, or cardiac angioplasty or stenting, or baseline electrocardiographic evidence of myocardial infarction.
Diabetes	Fasting glucose ≥126 mg/L (or a glucose ≥200 mg/L for those not fasting) or participant reported use of insulin or oral hypoglycemic agents.
Deep Vein Thrombosis	Participant reported history of deep vein thrombosis.
Dyslipidemia	Low-density lipoprotein cholesterol >130 mg/dL or participant reported use of lipid lowering medications.
Hypertension	Systolic blood pressure ≥140 mm Hg, diastolic blood pressure ≥90 mm Hg, or participant reported antihypertensive agent use.
Myocardial Infarction	Participant reported history of myocardial infarction or baseline electrocardiographic evidence of myocardial infarction.
Obesity	{Waist circumference [>102 cm for males or >88 cm for females]} or {body mass index ≥30 kg/m^2^}.
Peripheral Artery Disease	Participant reported history of lower extremity arterial bypass or leg amputation.
Stroke	Participant reported history of stroke or transient ischemic attack.

**Table A2:** Odds ratios * (ORs) and associated 95% confidence intervals (CIs) for the association between the preceding 30-day and one-year mean particulate matter 2.5 and incident respiratory infection related sepsis. Among 776 total events.

	Unadjusted	Model 1 ^a^	Model 2 ^b^	Model 3 ^c^
	Preceding 30-Day Mean OR (95% CI)			
**Particulate Matter 2.5**				
Per SD µg/m^3^ increase (SD** = 3.04)	1.01 (0.91, 1.12)	0.99 (0.89, 1.11)	1.00 (0.90, 1.12)	0.99 (0.87, 1.12)
*p*-value	0.87	0.90	0.98	0.84
**Particulate Matter 2.5**				
1st Quartile (3.27–10.53 µg/m^3^)	Referent	Referent	Referent	Referent
2nd Quartile (10.53–11.97 µg/m^3^)	1.18 (0.93, 1.49)	1.12 (0.88, 1.43)	1.16 (0.90, 1.49)	1.16 (0.87, 1.54)
3rd Quartile (11.97–13.78 µg/m^3^)	0.91 (0.71, 1.17)	0.85 (0.65, 1.10)	0.87 (0.66, 1.14)	0.85 (0.63, 1.14)
4th Quartile (13.78–28.11 µg/m^3^)	1.06 (0.81, 1.40)	1.04 (0.78, 1.38)	1.09 (0.81, 1.47)	1.04 (0.75, 1.42)
*p*-value_trend_	0.17	0.16	0.13	0.18
	**Preceding One-Year Mean OR (95% CI)**			
**Particulate Matter 2.5**				
Per SD µg/m^3^ increase (SD** = 1.73)	1.02 (0.94, 1.12)	0.99 (0.90, 1.09)	0.99 (0.90, 1.09)	0.98 (0.88, 1.09)
*p*-value	0.62	0.80	0.78	0.67
**Particulate Matter 2.5**				
1st Quartile (5.88–11.48 µg/m^3^)	Referent	Referent	Referent	Referent
2nd Quartile (11.48–12.58 µg/m^3^)	1.00 (0.79, 1.26)	0.91 (0.71, 1.17)	0.87 (0.67, 1.13)	0.83 (0.62, 1.11)
3rd Quartile (12.58–13.53 µg/m^3^)	1.02 (0.80, 1.29)	0.92 (0.72, 1.19)	0.95 (0.73, 1.23)	0.92 (0.68, 1.23)
4th Quartile (13.53–20.18 µg/m^3^)	1.03 (0.80, 1.32)	0.93 (0.72, 1.22)	0.91 (0.69, 1.20)	0.85 (0.63, 1.16)
*p*-value_trend_	1.00	0.89	0.77	0.60

* Estimated using Conditional Logistic Regression. ** Standard Deviation (SD). ^a^ Additionally adjusted education, income, region, temperature (30-day or 365 depending on pollution exposure), and urbanicity. ^b^ Additionally adjusted for tobacco and alcohol use. ^c^ Additionally adjusted for baseline health conditions.
